# Influence of penicillin treatment of horses with strangles on seropositivity to *Streptococcus equi* ssp. *equi*‐specific antibodies

**DOI:** 10.1111/jvim.15668

**Published:** 2019-11-26

**Authors:** John Pringle, Emma Storm, Andrew Waller, Miia Riihimäki

**Affiliations:** ^1^ Department of Clinical Sciences, Faculty of Veterinary Medicine and Animal Science Swedish University of Agricultural Sciences Uppsala Sweden; ^2^ Animal Health Trust Newmarket United Kingdom

**Keywords:** antibiotics, antigen A, antigen C, equine, iELISA, infection

## Abstract

**Background:**

Antibiotic treatment of horses with strangles is reported to impair the development of immunity to subsequent exposure to *Streptococcus equi* ssp *equi* (*S. equi*). However, apart from a single clinical report, evidence‐based studies for this hypothesis are lacking.

**Hypothesis/Objective:**

To determine whether penicillin treatment during clinical strangles influences the development or persistence of seropositivity to *S. equi*‐specific antibodies.

**Animals:**

A natural outbreak of strangles with 100% morbidity in 41 unvaccinated mature Icelandic horses.

**Methods:**

A prospective longitudinal study of acute clinical strangles from onset through full recovery approximately 10 months after the index case. Horses were monitored clinically 6 times for *S. equi*, as well as serologically for antibodies to antigens A and C of *S. equi* using an enhanced indirect ELISA. Seven horses received penicillin within 11 days of onset of fever (Group 1), 5 between 16 and 22 days after onset of fever (Group 2), and the remainder (Group 3, n = 29) received no antibiotics during clinical disease. The proportions of seropositive horses in each group were compared using an extension of Fisher's exact test with *P* < .05 as the level of significance.

**Results:**

Although all horses were seropositive to *S. equi* within 2 months of the index case, significantly fewer horses treated early (Group 1) remained seropositive by 4 to 6 months (*P* = .04 and .02, respectively).

**Conclusions and Clinical Importance:**

Findings support earlier suggestions that penicillin administered during acute strangles can interfere with persistence of humoral immunity to *S. equi*.

**Abbreviations**
Ag A: antigen A (SEQ_2190 of *S. equi*)
Ag C: antigen C (SeM of *S. equi*)
CI: confidence interval
CS: Clinical score
NL: nasopharyngeal lavage
PC: penicillin

## 1 INTRODUCTION

Strangles is a highly infectious upper respiratory tract disease in horses caused by the β‐hemolytic Lancefield group C bacteria, *Streptococcus equi* subspecies *equi* (*S. equi*). Acutely, the disease is associated with fever, lethargy, and swollen abscessed lymph nodes over a period of weeks to months,^1^, ^2^ after which most horses recover uneventfully.

Penicillin is the drug of choice for treatment of streptococcal infections in horses.^3^ However, there is controversy about whether to treat acutely infected horses with antibiotics or not,^2^ because most cases of strangles resolve without specific treatment.^4^ Nonetheless, recent reports suggests that, in some practice settings, the majority (78%) of horses with strangles receive some form of antibiotic treatment.^5^ However, administration of antibiotics to horses with clinical strangles may simply delay the maturation of abscesses, or may increase the risk of developing metastatic or “bastard” strangles.^2^ Furthermore, despite limited studies,^6^ administration of penicillin to horses in early stages of strangles or as prophylaxis has been suggested to interfere with the development of protective immunity and thereby render such animals susceptible to reinfection.^2^ When penicillin first became available to the medical profession, similar concerns about its impairment of the immune response arose. Specifically, as related to treatment of streptococcal infections in people,^7^ increased recurrence of infection was observed in human patients with scarlet fever (group A *Streptococcus*) treated with penicillin particularly early after the onset of clinical disease. Later work substantiated these observations,^8^ with a suggested mechanism being that very early administration of penicillin interfered with the humoral immune response against *Streptococcus*.^9^ Although humoral responses against *S. equi* correlate with protection,^10^ to the best of our knowledge, no evidence‐based studies document the effect of early penicillin treatment of strangles on the subsequent serologic response to *S. equi*.

A strangles outbreak with 100% morbidity occurred in a closed group of 41 mature Icelandic riding horses. Although antibiotic use was restricted during the clinical phase of the outbreak, because of the severity clinical signs or pressure from the owners, or both, 12 of the horses were prescribed courses of penicillin. It was hypothesized that penicillin treatment in clinical strangles would impair the development or long‐term persistence of antibodies specific to *S. equi*.

## 2 MATERIAL AND METHODS

### 2.1 Horses included in the study

A strangles outbreak with 100% morbidity occurred in late April 2015 in a closed group of 43 adult Icelandic horses. Two horses were euthanized at the onset of the outbreak because of the development of clinical signs of strangles in combination with advanced age and poor dentition. The remaining 41 horses (mean age, 16.0 ± SD 6.6 years), including 32 geldings and 9 mares, were followed prospectively clinically, serologically and by upper respiratory microbiology from acute illness through clinical recovery as part of a larger study on the development of silent carriers.^11^ All horses were *S. equi* positive by culture, qPCR positive, or both during the acute phase (first 7 weeks after the index case) and all but 1 horse had an episode of fever during that time. Because all horses were clinically affected during this outbreak, no isolation of clinically diseased horses was carried out and the same stable personnel handled all horses in the yard. When outdoors, horses were held in smaller groups based on sex, but sharing of water buckets was possible. In the stable, all horses had individual boxes with low walls that allowed nose‐to‐nose contact with horses from other paddock groups. Three horses were lost for final sampling 10 months after the index case (see below, clinical sampling), with 2 horses euthanized; 1 because of laminitis and the other because of peritonitis. The remaining horse was moved to another farm after being determined to be *S. equi* negative on nasopharyngeal lavage (NL).

### 2.2 Clinical sampling

**Figure 1:**
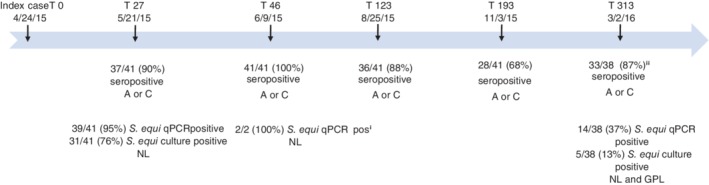
Time scale for sampling of the strangles outbreak in 41 mature Icelandic horses, with results of serology to antigen A (A) and antigen C (C) of an enhanced ELISA and testing for presence of *Streptococcus equi* from acute disease to full clinical recovery. ^i^Resampling of the sole 2 horses PCR negative at T27. ^ii^Three lost to follow‐up sampling T313: 2 euthanized and 1 moved off farm. For *S. equi* recovery nasopharyngeal lavage (NL) and guttural pouch lavage (GPL)

Data included here are from 5 herd visits, hereafter denoted serum sampling days post index case (T = 0) as T27, T46, T123, T193, and T313. Samplings T27 and T46 took place during acute clinical illness, approximately 4 and 7 weeks after the index case, and samplings T123‐T313 were conducted after clinical signs had resolved (approximately 4, 6, and 10 months after the index case; Figure 1). On each sampling occasion, all horses had complete physical examinations with clinical scoring assigned according to a modification of a previous scoring scheme^4^ (Table 1). Nasopharyngeal lavages were performed on all horses on T27, and T123‐T313, and on 2 horses on T46 (the 2/41 horses that were *S. equi* negative on the first sampling occasion). Guttural pouch lavages for *S. equi* also were conducted on T313 on all horses remaining on the premise. During the entire study, personnel used disposable protective clothing and changed gloves between horses. Nasopharyngeal lavage was performed as previously described^12^ by instilling 250 mL 0.9% NaCl via a foal feeding tube (Vycom REF 310.12) at the level of the nasopharynx with recovered fluid collected in a disposable plastic bag held over the nares and then transferred to sterile 50 mL plastic tubes (Sarstedts REF 547.004). All samples were stored at 4°C and on the following day analyzed for *S. equi* by q‐PCR.^13^

**Table 1:** Clinical scoring scheme for clinical signs of acute strangles, as modified from Tscheschlok^4^

Observation	Grade/cutoff	Score
Rectal temperature	≤38.2°C (100.8 F)	0
	>38.2°C (100.8 F)	1
Nasal discharge	None	0
	Serous	1
	Seromucoid	2
	Purulent	3
Lymph node swelling	None	0
	Mild	1
	Moderate/severe	2
	Abscess	3
Maximal score		7

### 2.3 Serology

Serum samples were stored at −20°C for subsequent serological analysis using an optimized indirect ELISA^14^ that targets both antigen A (SEQ_2190) and antigen C (SeM) of *S. equi* (Ag A and Ag C). Sera with iELISA optical density 450 nm (od 450) values ≥0.5 for the respective antigens were deemed seropositive.

### 2.4 Treatment

Of the entire group, 12 of 41 horses were treated with penicillin within the first 2 months after the index case. Reasons for penicillin treatment in the acute phase included decreased appetite, high or persistent fever, and lethargy, combined with owner pressure regarding horses with prolonged or worsening clinical abnormalities. Seven horses, including 6 geldings and 1 mare (median age, 15.7 years) were treated within the first 11 days after developing fever (Group 1) and for 7 to 17 days. The remaining 5 horses, all geldings and median age 14 years, were treated beginning 16 and 43 days after initial fever (Group 2) and for 5 to 54 days. Treatment included 20 mg/kg (33 340 IU/kg) procaine penicillin (Penovet vet, Boehringer Ingelheim Animal Health, Copenhagen, Denmark) IM q24h for 4 and 3 horses in Groups 1 and 2, respectively, and q12h for the remainder of horses in these groups. Three horses in each treatment group received an additional course of 20 mg/kg benzyl penicillin (Geepenil vet Orion Pharma Animal Health, Danderyd, Sweden) IV q8h for 7 to 10 days (Table 2). The remaining horses (Group 3), including 4 mares and 25 geldings (median age, 14.4 years), received no treatment apart from short courses of meloxicam (Metacam, Boehringer Ingelheim Animal Health) or flunixin meglumine (Flunixin N‐vet, Norbrook Laboratories Ltd, Newry, Northern Ireland) for relief of fever and lethargy.

Animal care approval for the study was obtained from the regional animal ethics committee.

### 2.5 Statistics

Descriptive statistics were calculated and comparisons between groups conducted using the Freeman‐Halton extension of the 2‐tailed Fisher exact probability test or the Wilcoxon ranked sign test. The level of significance was set at *P* < .05.

## 3 RESULTS

All horses were qPCR positive for *S. equi* on NL samples on sampling T27 (39/41 positive horses) or sampling T46 (2/2). Most of the horses (31/41; 76%) also were *S. equi* positive on culture at sampling T27 (Figure 1).

Untreated horses and those treated with penicillin did not differ in age, and all but 1 horse in Group 3 were initially positive for *S. equi*. Although clinical scores were not significantly different (*P* = .12‐1.0) those treated after 16 days of fever had numerically higher clinical scores (Table 2) at sampling T27. By sampling T46, clinical severity scores were lower and similar in all groups.

**Table 2:** Penicillin (PC) treatment^a^ timing and duration in days during acute strangles in 41 horses in relation to age in years (y), clinical scores (CS) and *Streptococcus equi* recovery during acute clinical strangles and after full recovery 10 months after the index case. Group 1 treated with PC within 11 days (d) of fever, Group 2 treated with PC between 16 and 43 days of onset of fever, and Group 3 received no antibiotics during acute clinical strangles

Group	Age Mean (range)	Fever to first PC mean (range)	Treatment duration mean (range)	Sampling period				
				T27	T46		T313	
				Clinical score: mean (95% CI)	*S. equi* +ve		Clinical score; mean (95% CI)	*S. equi* +ve
					Culture	PCR		
1 (n = 7)	15.7 y^b^ (7‐24)	7.7 d (3‐11)	11d (7‐19)	3.0^d^ (1.3‐4.7)	5/7	7/7	1.9^f^ (0.7‐3.0)	2/5^g^
2 (n = 5)	14 y^b^ (9‐24)	32 d (16‐43)	23 d (5‐54^c^)	4.4^d^ (3.0‐5.8)	5/5	5/5	1.4^f^ (0.0‐2.9)	2/7^g^
3 (n = 29)	14.4 y^b^ (7‐27)	na	na	3.2^d^ (2.5‐3.8)	21/28^e^	27/28^e^	1.9^f^ (1.3‐2.4)	10/26^g^

Abbreviation: na, not applicable.

a Three in each Group 1 and 2 included both IM procaine and periods of crystalline penicillin IV.

b *P* = .84‐1.0, Wilcoxon ranked sign.

c Fifty‐four days treatment of one horse with suspected intra‐abdominal abscess, one treated for traumatic limb wound unrelated to strangles.

d Group 1 to 2 *P* = .12, Group 2 to 3 *P* = .12, Group 1 to 3 *P* = 1, Wilcoxon ranked test.

e One horse not sampled.

f Group 1 to 2 *P* = 1.0, Group 2 to 3 *P* = .50, Group 1 to 3 *P* = .75, Wilcoxon ranked test.

g *P*_B_ = .99 Freeman‐Halton extension of the Fishers exact test, where *P*_B_ is the probability that the null hypothesis holds.

Within 7 weeks (T46) after the index case, all horses had seroconverted to Ag A (Table 3), 32/41 (78%) had seroconverted to Ag C (Table 4), and thus all (41/41) had converted to either antigen A or C (Table 5). However, approximately 4 months after the index case (T123), significantly fewer horses in Group 1 remained seropositive to Ag A (*P* = .03; Table 3) and to either Ag A or C (*P* = .04; Table 5). By 7 months (T193), significantly fewer horses are in Group 1 compared to Group 2, and 3 remained seropositive to Ag A (*P* = .04) and either Ag A or C (*P* = .02; Tables 3 and 5). Overall, once acute clinical strangles had resolved (sampling T123‐T313), horses in Group 1 had a lower proportion of animals that remained seropositive to either Ag A or Ag C (Tables 3, 4, 5). Of note, at T313, 14/38 were determined to be clinically silent carriers, but the proportion of *S. equi* positives between these groups did not differ (*P* = .99; Table 2).

**Table 3:** Proportion and percentage (in brackets) of horses positive for *Streptococcus equi* antigen A^14^ over time following a strangles outbreak with 100% morbidity in 41 adult Icelandic horses. Group 1: penicillin administered ≤11 days after onset fever; Group 2: penicillin administered ≥16 days after onset fever; and Group 3: no antibiotics during acute strangles

Group	Sampling T 27	Sampling T 46	Sampling T 123	Sampling T 193	Sampling T 313
Group 1	7/7^a^ (100%)	6/7^b^ (86%)	3/7^c^ (43%)	2/7^d^ (29%)	3/7^e^ (43%)
Group 2	5/5^a^ (100%)	5/5^b^ (100%)	5/5^c^ (100%)	5/5^d^ (100%)	3/5^e^ (60%)
Group 3	25/29^a^ (86%)	29/29^b^ (100%)	24/29^c^ (83%)	19/29^d^ (66%)	21/26^e^ (81%)
All horses	37/41	40/41	32/41	26/41	27/38

*Notes*: Groups 1 versus 2 versus 3: ^a^ *P*_B_ = .75, ^b^ *P*_B_ = .29, ^c^ *P*_B_ = .03, ^d^ *P*_B_ = .04, ^e^ *P*_B_ = .08. Freeman‐Halton extension of the Fisher exact probability test where *P*_B_ is the probability that the null hypothesis holds.

**Table 4:** Proportion and percentage (in brackets) of horses seropositive for antigen C^14^ over 10 months following a strangles outbreak with 100% morbidity in 41 adult Icelandic horses. Group 1: penicillin administered ≤11 days after onset fever; Group 2: penicillin administered ≥16 days after onset fever; and Group 3: no antibiotics during acute strangles

Group	Sampling T 27	Sampling T 46	Sampling T 123	Sampling T 193	Sampling T 313
Group 1	2/7^a^ (29%)	5/7^b^ (71%)	2/7^c^ (29%)	0/7^d^ (0%)	2/7^e^ (29%)
Group 2	3/5^a^ (60%)	5/5^b^ (100%)	4/5^c^ (80%)	3/5^d^ (60%)	4/5^e^ (80%)
Group 3	13/29^a^ (45%)	22/29^b^ (76%)	14/29^c^ (48%)	6/29^d^ (21%)	15/26^e^ (58%)
All horses	18/41	32/41	20/41	9/41	21/38

*Notes*: Groups 1 versus 2 versus 3: ^a^ *P*_B_ = .62, ^b^ *P*_B_ = .61, ^c^ *P*_B_ = .20, ^d^ *P*_B_ = .05, ^e^ *P*_B_ = .23. Freeman‐Halton extension of the Fisher exact probability test where *P*_B_ is the probability that the null hypothesis holds.

**Table 5:** Proportion and percentage (in brackets) of horses seropositive for either antigen A or C of *S. equi* 14 over 10 months following a strangles outbreak with 100% morbidity in 41 adult Icelandic horses. Group 1: penicillin administered ≤11 days after onset fever; Group 2: penicillin administered ≥16 days after onset fever; and Group 3: no antibiotics during acute strangles

Group	Sampling T 27	Sampling T 46	Sampling T 123	Sampling T 193	Sampling T 313
Group 1	7/7^a^ (100%)	7/7^b^ (100%)	4/7^c^ (57%)	2/7^d^ (29%)	4/7^e^ (57%)
Group 2	5/5^a^ (100%)	5/5^b^ (100%)	5/5^c^ (100%)	5/5 ^d^ (100%)	5/5^e^ (100%)
Group 3 (no	25/29^a^ (86%)	29/29^b^ (100%)	27/29^c^ (93%)	21/29^d^ (72%)	24/26^e^ (92%)
All horses	37/41	41/41	36/41	28/41	33/38

*Notes*: Groups 1 versus 2 versus 3: ^a^ *P*_B_ = .75, ^b^ *P*_B_ = 1.0, ^c^ *P*_B_ = .04, ^d^ *P*_B_ = .02, ^e^ *P*_B_ = .06. Freeman‐Halton extension of the Fisher exact probability test where *P*_B_ is the probability that the null hypothesis holds.

## 4 DISCUSSION

These results provide supportive evidence that antibiotic treatment during acute clinical strangles can impair persistence of humoral immunity against *S. equi*. Based on principles of immunology, these findings were not unexpected. However, even the delay of penicillin administration on average of >7 days after the onset of fever still influenced the duration of the serologic response. Nonetheless, the findings strengthen the evidence for strangles treatment impairing humoral immunity whereas previous suggestions relied solely on a single case report.^6^ Variations in the clinical severity of strangles may have confounded our findings. At the onset of this outbreak and based on current literature,^1^ antibiotic treatment of clinically affected horses was discouraged. Unfortunately, although antibiotics were administered to some more severely affected horses, others likely were treated with antibiotics because of pressure from the owners rather than medical indication. Moreover, based on clinical scores, horses treated with antibiotics during acute clinical strangles in general had similarly severe clinical signs of strangles as did those not treated with antibiotics, and thus likely reacted to an infective dose of *S. equi* sufficiently high enough to induce disease and trigger a robust immune response.^15^

Other potential confounding factors include whether the group had previous exposure to strangles, and if exposure to *S. equi* in this outbreak was restricted to only some members of the group. Because we had no pre‐exposure serology results, it is unknown whether some of the horses on the premise might have been seropositive to *S. equi* at the outset of the outbreak. However, the premise was a closed stable with most of the animals born and raised on the farm and with no history of previous disease suggestive of strangles. Thus, it is likely that most were serologically naive. Furthermore, because exposure to *S. equi* has been shown to protect against clinical strangles,^10^ previous exposure to *S. equi* in these horses would be inconsistent with a morbidity rate of 100%. Moreover, all horses were shown to be *S. equi* positive on NL during the clinical phase of the outbreak, with the majority (31/41) being bacterial culture positive (Table 2 and Figure 1). These findings, in combination with seropositivity to either Ag A or Ag C by the first 7 weeks of the outbreak (Table 5), indicate an active infection by *S. equi* in all horses during this time.

Strikingly, by approximately 4 months after the index case (T123), fewer horses treated with penicillin earlier (Group 1) remained seropositive (Table 5), suggesting a more rapid waning of humoral immunity in these animals. Similarly, studies in people have shown that early penicillin treatment of streptococcal infections may interfere with the development of humoral immunity^8^, ^9^ or increase the risk of recurrence of clinical disease at a later date.^7^ A key difference between these studies in humans and the strangles outbreak described here was administration of penicillin within a few days of infection in the human patients versus a mean of 7.7 days in the horses. Thus, our data suggest that immediate^2^ or prophylactic^6^ antibiotic treatment of strangles, although potentially curative, likely would markedly impair the development of humoral immunity to *S. equi*, leaving such treated animals susceptible to clinical disease from future challenges.^10^

Without continued immune stimulation, antibody responses wane over time. Thus, it was unexpected to find that some animals, in particular those in Group 1, were once again seropositive by the final sampling (T 313; 10 months after the index case). At that time, more than 33% of the horses (14/38) were identified as long‐term silent carriers.^16^ However, silent carrier status was not associated with differences in seropositivity to *S. equi* among groups, because the proportion of long‐term silent carriers was similar in all 3 groups (Table 2). Although the premises were closed to horse movement, for practical reasons resident horses were grouped together on the same paddock or summer pastures. Thus, nose‐to‐nose contact likely allowed interanimal transmission of *S. equi*, as suggested by sequencing studies.^16^ Therefore, reinfection by silent carriers may have been key factor for those animals regaining seropositivity at the point of final sampling (Tables 3, 4, 5).

## 5 CONCLUSION

Treatment of horses with penicillin during acute clinical strangles is associated with persistence of seropositivity to *S. equi*. Whether this limitation in the duration of antibodies against *S. equi* leads to increased susceptibility to recurrence of clinical strangles after re‐exposure to an infective challenge of *S. equi* remains to be determined.

## CONFLICT OF INTEREST DECLARATION

Authors declare no conflict of interest.

## OFF‐LABEL ANTIMICROBIAL DECLARATION

Authors declare no off‐label use of antimicrobials.

## INSTITUTIONAL ANIMAL CARE AND USE COMMITTEE (IACUC) OR OTHER APPROVAL DECLARATION

This study was approved by the Swedish Ethical Committee on Animal Experiments (diary nr C 36/14). All horse owners provided informed consent for use of their animals in the study.

## HUMAN ETHICS APPROVAL DECLARATION

Authors declare human ethics approval was not needed for this study.
